# Many-Scale Investigations of the Deformation Behavior of Polycrystalline Composites: I—Machine Learning Applied for Image Segmentation

**DOI:** 10.3390/ma15072486

**Published:** 2022-03-28

**Authors:** Yanling Schneider, Vighnesh Prabhu, Kai Höss, Werner Wasserbäch, Siegfried Schmauder, Zhangjian Zhou

**Affiliations:** 1 Institute for Materials Testing, Materials Science and Strength of Materials (IMWF), University of Stuttgart, Pfaffenwaldring 32, D-70569 Stuttgart, Germany; viggu9460@gmail.com (V.P.); siegfried.schmauder@imwf.uni-stuttgart.de (S.S.); 2 Graduate School of Excellence advanced Manufacturing Engineering (GSaME), University Stuttgart, Nobelstr. 12, D-70569 Stuttgart, Germany; kai.hoess@gsame.uni-stuttgart.de; 3 Institute of Materials Science, University of Stuttgart, Heisenbergstraße 3, D-70569 Stuttgart, Germany; werner.wasserbaech@imw.uni-stuttgart.de; 4 School of Materials Science and Engineering, University of Science and Technology Beijing, Beijing 100083, China; zhouzhj@mater.ustb.edu.cn

**Keywords:** machine learning, digital image segmentation, ground truth, denoising, size spectrum

## Abstract

Our work investigates the polycrystalline composite deformation behavior through multiscale simulations with experimental data at hand. Since deformation mechanisms on the micro-level link the ones on the macro-level and the nanoscale, it is preferable to perform micromechanical finite element simulations based on real microstructures. The image segmentation is a necessary step for the meshing. Our 2D EBSD images contain at least a few hundred grains. Machine learning (ML) was adopted to automatically identify subregions, i.e., individual grains, to improve local feature extraction efficiency and accuracy. Denoising in preprocessing and postprocessing before and after ML, respectively, is beneficial in high quality feature identification. The ML algorithms used were self-developed with the usage of inherent code packages (Python). The performances of the three supervised ML models—decision tree, random forest, and support vector machine—are compared herein; the latter two achieved accuracies of up to 99.8%. Calculations took about 0.5 h from the original input dataset (EBSD image) to the final output (segmented image) running on a personal computer (CPU: 3.6 GHz). For a realizable manual pixel sortation, the original image was firstly scaled from the initial resolution 1080${}^{2}$ pixels down to 300${}^{2}$. After ML, some manual work was necessary due to the remaining noises to achieve the final image status ready for meshing. The ML process, including this manual work time, improved efficiency by a factor of about 24 compared to a purely manual process. Simultaneously, ML minimized the geometrical deviation between the identified and original features, since it used the original resolution. For serial work, the time efficiency would be enhanced multiplicatively.

## 1. Introduction

Accompanying the progressing development of computer technology, the amounts of data that have to be processed are exploding. This increase in data is driving the search for better automated data sortation and data handling solutions. The new research branches of big-data-driven sciences that deal with such enormous amounts of data are gaining in importance. Besides improving data pipelines’ efficiency, organizations desire to gain additional knowledge or relationships from their data with ease. To achieve this goal, the algorithms need to perform their tasks similarly to a human being and make decisions based on learning from experience and analytic information. Data science is also referred to as “the fourth paradigm of science” [1]. In materials science, the study of such “intelligent algorithms” is considered “material informatics”. For autonomous data parsing and simultaneous learning from the received data, machine learning (ML) provides a nearly endless application range, e.g., in medical analysis, business trend prediction, industrial engineering, and materials science.

The concept of ML can be traced back to the 1950s [2]. In the 1980s, ML developed into an independent discipline and became a core element in data science [3]. Data mining is an important application of ML, which can extract and leverage hidden correlations and important relationships among large amounts of data. The user’s eyes can hardly observe such correlations. Despite its rapid development, ML is still in the formative stage. This means many factors of ML are not fully fixed yet. Possibly, this is due to the vast application potentials of ML and the large number of ML methods/techniques. Still, there are no unified ML classifications in the literature [3,4,5]. Characteristics of ML can be summarized as (i) autonomous (executing tasks); (ii) requiring no explicit programming; (iii) able to parse data; (iv) able to learn from data; (v) able to make decisions (delivering results); (vi) able to transfer the learned “knowledge” as solutions for further problems. Just as there is no set ML definition, no standardized classification categories exist for ML. According to [6], ML can be categorized into unsupervised, supervised, and reinforcement learning. The last execution is contingent on “learning” experience—i.e., it depends on previously classified results. Following [7,8], semi-supervised learning is also an accepted classification. In these ML algorithms, the training data are partly labeled and partly not [9], whereby the amount of unlabeled data often exceeds the labeled data. According to the characteristics of algorithms for implementing ML methods, they can be divided into two types: (i) shallow learning methods, mainly for cases requiring manual feature extraction and linear classification, e.g., support vector machine, decision tree, and naive Bayes classifier; (ii) deep learning methods, which are suitable for the automatic feature extraction and nonlinear classification, e.g., convolutional neural networks (CNN) and recurrent neural networks (RNN). Recently, U-Net [10] showed the ability to detect detailed microstructural features with low training data requirements. U-Net is a deep-learning CNN and applies a reinforcement learning algorithm to solve, e.g., supervised learning problems. Among all the available ML algorithms, the neural networks currently receive the most attention in research. Nearly 40 types of neural networks are available, e.g., backpropagation networks, perceptrons, self-organizing maps, the Hopfield network, and Blotzmann machines [9]. The artificial neural network (ANN) is an important one in the neural network family and has applications in many fields, such as pharmaceutics, traffic management, materials science, and engineering. It belongs to the nonlinear processing class and is adaptive. ANNs are inspired by the biological neural networks found in animal brains. The neurons possess a layer-to-layer arrangement, and the data propagate from one layer to another during the training phase. Three types of layers can be identified: the input layer, the hidden layers, and the output layer. One or multiple hidden layer(s) can be used to solve nonlinear problems. The reader may refer to [11] for a more comprehensive review of ANNs. Many review articles about ML have been published. Wei et al. [9] presented a summary of ML in materials science, and Butler et al. for molecular and materials science. Ramprasad et al. [12] reported the recent applications and prospects of ML in materials informatics. A review of the applications of ML and data mining approaches in continuum materials mechanics can be found in Bock et al. [13]. Data fusion is the main functionality of ML. A survey on ML for data fusion is given in Meng et al. [14].

Image segmentation is one of the application branches of ML in materials informatics. Image segmentation in itself is a study field, which means that image segmentation is independent of ML. Among various (digital) image processing techniques, image segmentation plays a vital role in analyzing the given image. Segmentation refers to the process of identifying and isolating the surface and regions of the digital image corresponding to the structural units [4]. According to [4], segmentation algorithms are mainly based on two basic properties: discontinuity and similarity. Similarly to ML, there is no unified classification of image segmentation methods. Jaiswal and Pandey [15] categorized them into supervised segmentation and unsupervised segmentation methods. Kannan et al. [4] lists four segmentation categories: edge detection, thresholding, region-based, and feature-based. Reviews of image segmentation can be found, e.g., in [4,15,16,17,18,19,20,21]. Some review reports concentrate on medical imaging, such as [22,23,24,25].

The current work applied ML methods to segment electron backscatter diffraction (EBSD) images for polycrystalline microstructures containing no less than 500 grains. An autonomous process should significantly improve the time efficiency of input data preparation for finite element (FE) simulations, in which representative polycrystalline real microstructures were used. ML also benefited the detailed feature extraction to a great extent. Local morphological characteristics could be better mapped for FE predictions. Our process of image segmentation includes three stages: preprocessing, ML classification, and postprocessing. The three supervised ML methods—decision tree, random forest, and support vector machine—were adopted, and their accuracies were compared. All achieved satisfying results. With freely available Python packages, a self-coded ML algorithm was developed. Our EBSD images possessed an original resolution of 1080 × 1080 pixels for an 80 × 80 μm${}^{2}$ sized sample. On a personal computer (PC) with a central processing unit (CPU) operating at 3.6 GHz, our code processed each image in about 30 min. The data training for the ML took approximately two-thirds of the whole time. Our approach with the usage of ML required only about $\frac{1}{24}$ of the manual work time, and simultaneously allowed for much more detailed subregion identification regarding the morphological features. From the original input of data to finishing the geometrical adaptive meshing ready for FE calculations, the process took about 3–4 days, depending on the manual work intensity. This work contributes to autonomous segmentation for colored images, which is far less reported on than the same for monotonic images [21]. It supports the use of big-data-driven science in materials science, and supports the applicability of machine learning (new branch) to improving the efficiency of simulations (existing branch).

## 2. Materials and Experiments

Our work’s original goal was to experimentally and numerically investigate the deformation behavior of SnO${}_{2}$ oxide dispersion strengthened (ODS) Ag alloys in multi scales since any specific material behavior results from coupled mechanisms on different scales. Micromechanical FE simulation is an essential tool to investigate material deformation behaviors numerically. The microscale acts as a bridge, which links the mechanisms on the nanoscale and macroscale. Our study currently emphasizes predictions of the influence of Σ3-twins on the local deformation behaviors. In such calculations, the applied microstructure should be well representative to achieve correct predictions. During the data preparation for the FE calculation, the time-consuming and tedious work of the manual pixel sortation of real microstructures has led to the consideration of the image segmentation by using ML. The development of advanced ML algorithms shows the potential to replace manual classification. The ML methods and segmentation results presented in this report are the first part of our work. The second part deals with FE results and their comparison with experimental findings, which will be presented in another report. Wasserbäch and Skrotzki [26,27] presented most of the measured results.

### 2.1. Materials

We used six commercial composites with various oxide concentrations and sizes. The internal oxidation (IO) processes [28,29,30] manufactured three, and the powder metallurgical (PM) process [29] the other three. The hot extrusion process is the commonly applied treatment in the industrial production of the Ag/SnO${}_{2}$ metal matrix composites (MMCs). The usage of different SnO${}_{2}$ powders can control the final oxide sizes. Such MMCs are also a kind of functionally graded materials. Table 1 lists five material characteristic values for the two MMCs used in the current work and for the pure Ag as a comparison. For simplicity, only EBSD images will be shown in the following. Further descriptions of experimental results and their application in FE simulations are presented in the second part.

### 2.2. EBSD Images

**Figure 1:**
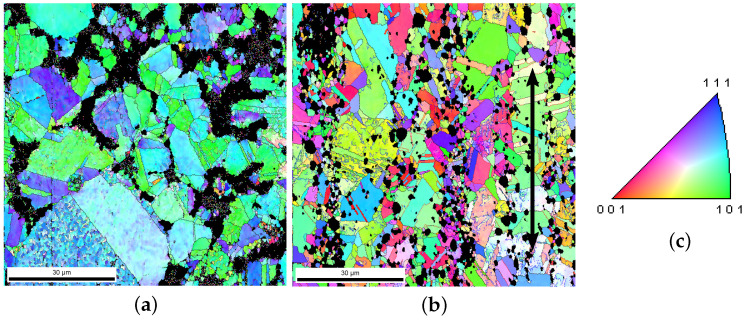
(**a**) The green body morphology of the PM12-2 composite before hot extrusion [26]; (**b**) the microstructure of PM12-2 in the longitudinal direction after hot extrusion (Table 1), where the arrow presents the extrusion direction; (**c**) grain orientation color code valid for the Ag phase of all the EBSD measurements for Ag/SnO${}_{2}$ MMCs.

**Figure 2:**
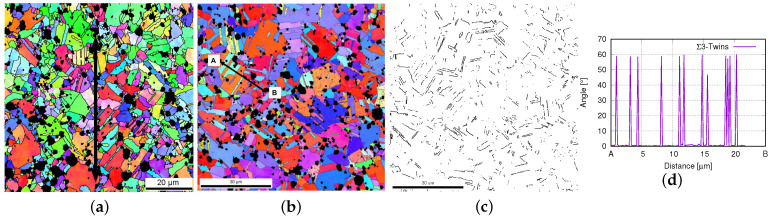
PM12-3 composite (Table 1) from EBSD measurements: (**a**) in the longitudinal direction, where the arrow presents the extrusion direction [26]; (**b**) in the transverse direction [26]; (**c**) the Σ3-twin boundaries for the microstructure given in (**b**); (**d**) the measured Σ3-twin angles along the line AB marked in (**b**).

Due to the shaping process, extraordinary distortions and recrystallizations appeared. The observed microstructures, including Σ3-twins, and textures, are due to recrystallization during or immediately after the hot-extrusion process (at high temperature). In our samples, the true strain partly reached the value of 500% caused by hot extrusion. The Ag phase possessed sharp textures after the extrusion. This shaping, leading to cross-section reduction, introduced tensile deformation of the material. Consecutively, tensile tests were performed with a loading direction identical to the extrusion one. Unexpectedly, the tension test caused a reduction in the texture sharpness. During tensile loading (cold-working), the SnO${}_{2}$ particles destroy the existing texture, including Σ3-twins. The experiment revealed that the texture intensity (around <001> fiber) and the volume fraction of twins are reduced. During hot extrusion, the Ag phase is very soft and the dislocations (in the Ag phase) can bypass the oxide particles. This means oxide particles’ effect on the texture evolution during hot extrusion is not as evident as during cold-working. Figure 1a denotes the material’s status in the green body of the PM12-2 composite (Table 1) before hot extrusion, and Figure 1b shows the material’s status after hot extrusion and before the tension test in the longitudinal direction. The arrows in Figure 1b and Figure 2a show the loading direction. Figure 1c is the color code shared by all the EBSD images for the Ag phase in Ag/SnO${}_{2}$ MMCs. The black areas in all EBSD images represent the SnO${}_{2}$ phase. Figure 2a,b illustrates the material’s status before tension and after the extrusion of the PM12-3 composite in the longitudinal and cross-sectional directions. Figure 2c shows the twin boundaries of Σ3-twins in Figure 2b. Figure 2d denotes the measured angles of Σ3-twins along the line AB marked in Figure 2b.

## 3. Machine Learning Applied for Image Segmentation

In the range of ML applied in microstructure processing (digital image segmentation), different works focus on various aspects concerning the microstructure. Furat et al. [31] semantically segmented the tomographic images by combining ML methods and conventional image processing steps. In their work, 2D and 3D U-Net [10] were used. Due to the poor resolution of tomographic images, 3D X-ray diffraction measurements were defined as ground truth during the data training. Pütz et al. [32] investigated advanced high-strength steels by using representative volume elements with periodic material structures using virtual (artificial) microstructures. Since the input parameters are the most critical part of generating the RVEs, an ML algorithm was trained to reproduce input data parameters equivalent to the real microstructural and morphological parameters. In order to characterize the critical events in microstructures, e.g., twin activity, Sharma et al. [33] explored the potential for informing the microscope’s observation strategy by using a decision tree (ML) model. Their resultant framework has been taken as the first step towards intelligent microscopy for the efficient observation of stochastic events during in situ microscopy campaigns. A methodology based on supervised learning was introduced to characterize and reconstruct stochastic microstructures [34]. In this work, the example microstructures were all two-phase ones. In integrated computational material engineering, two interesting topics are the coupling of computational thermodynamics and kinetics and process parameter optimization. To overcome the bottlenecks of the slow responses in kinetic calculations and the poor quality of a large amount of numerically predicted thermodynamic data, Li et al. [35] employed an unsupervised ML method to clean the data, which resulted in an extensive tabulated thermodynamic dataset. Consecutively, the extensive dataset’s parameterization was performed via artificial neural networks to achieve the nonlinear equation consisting of base functions and parameterization coefficients. In chemical engineering, Tercan et al. [36] found structural regions of interest in a complex phase diagram and identified local environments’ characteristics by coupling their own code with freely available ML algorithm packages. Tawfik et al. [37] presented an ML application to calculate the vibrational properties of crystals using quantum mechanical methods. Their complementary machine learning methods can rapidly and reliably recapitulate entropy, specific heat, effective polycrystalline dielectric function, and a non-vibrational property (bandgap). Baturynska et al. [38] advanced a conceptual framework which combines the FE simulation and ML to optimize process parameters for powder bed fusion additive manufacturing. The phenomenon of grain boundary phase transition is an emerging field that was until recently dominated by experiments. Atomistic modeling can predict interface structures and has the major bottleneck of a lack of computational tools. Based on evolutionary algorithms (unsupervised ML method), Ramprasad et al. [12] developed a computational tool to predict the structures of interfaces. Their tool can reveal new ground states and multiple grain boundary phases, where molecular dynamic simulations demonstrated the grain boundary phase transition.

In investigations of material’s behaviors, ML can be used independently and coupled with other methods, e.g., coupled with experiments, simulations, and manufacturing. The former case means ML is a step in the whole working process, and its results might be used by the following steps or as the final results, but there is no interchange or interaction between ML and other working steps. Our work belongs to this case. The segmented image resulting from ML serves as the microstructure for the FE meshing. No feedback from FE results would influence or modify the ML methods, also not necessary in this work. Pal et al. [21] mentioned that the autonomous segmentation for colored images had been much less reported than for monotonic images. Our work deals with the segmentation of Ag grains and SnO${}_{2}$ particles in colored EBSD images by using ML algorithms. The primary task is to extract the polycrystalline Ag grains, since they possess various colors. These lead to more complexity for the feature identification of Ag grains than for the black SnO${}_{2}$ particles. The input dataset (EBSD image) describes a complex local morphology. It is not the simple case with two phases described by two colors. Our problem belongs to the category of supervised learning.

This work aimed to efficiently parse the data (pixels in EBSD images) and further apply the segmentation result (microstructure with a single-pixel color for each grain) for the subsequent step investigation, e.g., meshing for FE simulation. Here, the pixel color was used to identify micromorphology and was irrelevant to grain misorientations and other data obtained from EBSD. This means the pixel sortation process did not change any measured properties, such as lattice orientations. The measured orientations can be applied as input data (for theory) in FE simulations.

### 3.1. Image Processing Steps and Preprocessing

**Figure 3:**
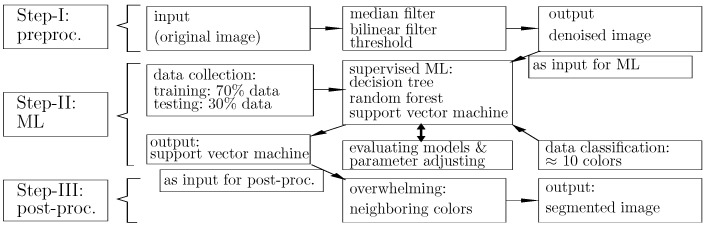
The workflow chat for the image segmentati on (preproc.: preprocessing, post-proc.: postprocessing).

EBSD images, as shown in Figure 1a,b and Figure 2a,b, provide the actual microstructure cut-outs in this work. All our EBSD images for Ag/SnO${}_{2}$ composites have a resolution of 1080 × 1080 = 1,166,400 pixels representing an 80 × 80 μm${}^{2}$ area. To achieve a good meshing quality and decrease the meshing process’s complexity, clearly distinguishable pixels are required for each subregion (per grain or particle/cluster). Distinguishable pixels in this context mean identical and individual pixel colors without noises for any individual subregion. Any original EBSD image contains noises, inevitable outliers, or disturbances, which frustrate pixel sortation into a meshed structure. Figure 3 presents the workflow of the image segmentation used in the current work. The segmentation process includes three steps, preprocessing (Step-I), ML (Step-II), and postprocessing (Step-III). Our Python algorithms include self-written code and inherent libraries/packages.

**Figure 4:**
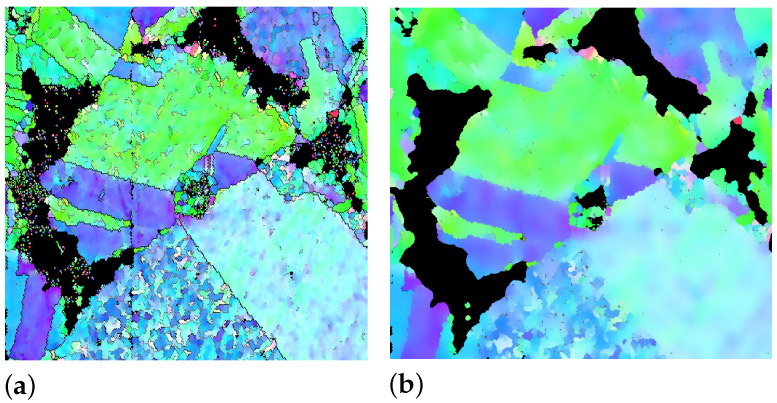
An example of data cleaning done for the original raw data in the preprocessing: (**a**) a cut-out of the original image in Figure 1a; (**b**) after the denoising.

**Figure 5:**
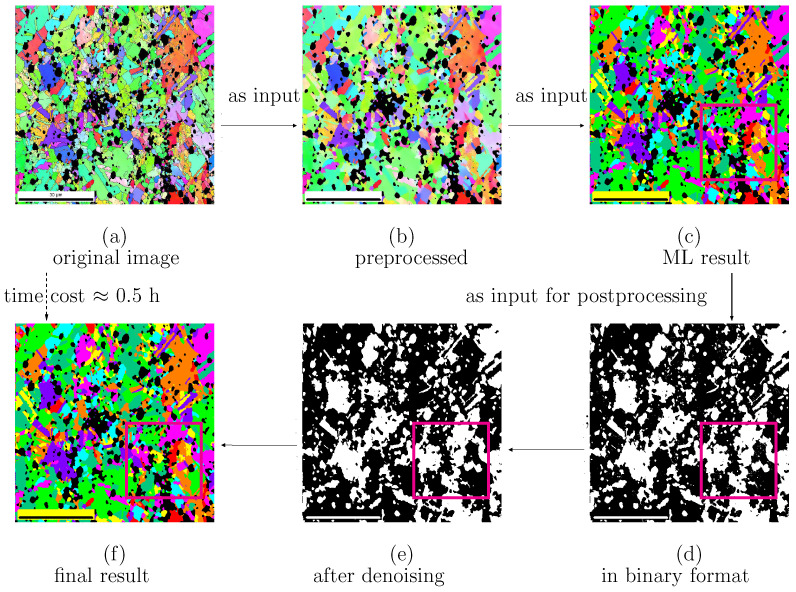
Autonomous image segmentation for MP12-2 Ag/SnO${}_{2}$ composite (Table 1): (**a**) original EBSD image; (**b**) resultant image after denoising by the preprocessing; (**c**) resultant image after the ML/random forest model; (**d**) monotonic image obtained by transforming (**c**) as preparation for denoising in postprocessing; (**e**) monotonic image after postprocessing; (**f**) final segmented color image after postprocessing.

With the collected data (the pixel colors in original EBSD images) at hand, the first step is preprocessing for the feature extraction. The data need to be prepared and cleaned due to outliers and disturbances, which could negatively affect the final result’s quality. Data cleaning partially removes the noise in the raw data to obtain suitable inputs for the model training in ML and is a crucial process of data preparation. Our collected data are given in red green blue (RGB) color scale. In the input image, various colors represent subregions of Ag grains and oxide particles. Two inherent Python libraries, “cv2 . medianBlur (image, 11)” and “cv2 . bilateralFilter (image, 25, 100, 100)”, denoise the black subregions and colored ones, respectively. Subsequently, the data were saved into an excel CSV file. As a contrast to noises, sample images manually cropped from the original image have dimensions of approximately 60 × 60 pixels for each color. The RGB values of each pixel in the sample image are written into the CSV file using Python’s CSV library. Corresponding target labels are given to the RGB values, also called the features. Using a CSV file for this purpose has several advantages. They (i) are relatively safe; (ii) can clearly distinguish between the numeric numbers and text numbers; (iii) do not manipulate data and store the data as they are; (iv) can also be opened by any conventional text editor; (v) read and import large CSV files faster and consume less memory than an excel file. As an example, Figure 4a presents a cut-out from the original EBSD image shown in Figure 1a. Figure 4b, as a comparison of the raw data in Figure 4a, illustrates cleaned data after the denoising in the preprocessing. The output of the data cleaning procedure denoted as an example in Figure 5b is the ML algorithm’s input and ready for the ML training algorithm.

### 3.2. Machine Learning

The second step of the image segmentation identifies subregions by using ML. The EBSD image acquisition (data visualizations) is the data collection for ML. The resulting data from the preprocessing described in Section 3.1 are prepared data, an example of which is shown in Figure 5b. The next step in the workflow would be choosing an appropriate ML model. Choosing the model depends on the type of problem at hand. It is important to know the major techniques of supervised and unsupervised learning to select suitable models. Most of the practical ML problems use supervised learning, for which the ML algorithm was trained to make user-defined correlations or classifications. In this case: (i) it needed a set of input variables {x} and a set of output variables {y}; (ii) ML methods approximated the relations between {x} and {y} using a learning algorithm. The objective was to generate a surrogate model function y = f(x) to calculate y for any new values of x. In other words, the algorithm was trained for labeled data (objective function y = f(x) and domain variables x are identified by the user). The algorithm made predictions on new data based on the training data. Training was stopped once the required accuracy of the surrogate model was reached. Supervised learning can be categorized into classification and regression. For the former, the output could possess distinctive classifications, e.g., “red”, “blue”, “green”, or “black”. If the algorithm divides the labeled input data into two classes, then it is a binary classification. The separation into multiple classes is called multi-class classification. For the regression, the output variable obeys a continuous numeric function depending on past trends or the correlation between x and y values gained from the training data. Krishna [39] lists some examples for supervised and unsupervised learning and compares their differences. A comparison of classification and regression models can be found in [40]. In unsupervised learning, the model predicts the output without being trained on a labeled dataset. These algorithms find an underlying pattern and the structure in the data and sort the data depending on similarities. The user does not explicitly train the model, but the model is expected to find compelling patterns and sort the data independently. Unsupervised learning can also be classified into the two categories, clustering and association, although many other divisions, such as anomaly detection, dimensionality reduction, and many more, exist. For a comparison of characteristics of supervised learning vs. unsupervised learning, the reader may refer to [39,41].

Our input data for ML were pixel colors resulting from preprocessing. The ML models aimed to identify the different colors and classify them into microstructural features with high accuracy. Since there was no hidden pattern that had to be extracted from the data and the total number of classes in the image was known in advance, it was expedient to apply a supervised learning algorithm. Actually, the clustering algorithms of unsupervised learning can also segment the pixels. However, ML has considerably lower accuracy than an algorithm using supervised data classification. Additionally, there is some difficulty in controlling the classification output, since there is no user interference in the learning phase for an unsupervised ML. Different ML algorithms are suitable for various specific problems. In our case, three supervised ML methods were adopted, decision tree, random forest, and support vector machine. For the decision tree, ML modeling in Python used iterative Dichotomizer 3 (ID3) and Gini index (Gini). ID3 utilizes entropy and information gain to construct the decision tree. It is beneficial to use it when the separation between output classes is slight, while many data samples are available [42]. The Gini index is suitable if the output classes are easily distinguishable by spatial separation. The implementation is computationally easier than ID3. Our Gini algorithm follows the procedures in [43]. The reader may refer to [42,43,44,45,46] for the detailed mathematic algorithms behind the three ML models mentioned above.

Besides the cleaned data from step I (data preparation), data classification is also required for ML. It is necessary to train the ML model regarding two factors: (i) what constitutes a particular color class; (ii) which color ranges can be classified into particular color classes. If the available amount of data is not sufficient, a risk of underfitting (biasing) the model can appear, which possibly deteriorates the output results. If the training data are sufficient to train the ML model, the user can proceed to add data labels or improve the existing data label. In our case, more than one million data samples were sufficient. Each pixel corresponds to a data sample. The output result shown in Figure 5d supports this conclusion. Multi-class classification is needed for the datasets in colored images. In our case, there are 9–10 classifications depending on individual image colors. It needs the so-called labeling to classify the data in a supervised algorithm. Additionally, important is to shuffle the training data randomly in order to minimize the chances of overfitting and false correlations. This improves the ML model’s output quality and the predictive performance of the model. In our supervised ML algorithm, a little more than 1.16 million datasets (1080 × 1080 pixels, each RGB pixel color one dataset) were stored in the CSV file. The entire dataset was divided into training and testing datasets. The ML model was: (i) trained to be sophisticated for parsing data by using the training dataset and (ii) tested for its accuracy by using the test dataset.

The algorithm should differentiate the aberrant colors and replace them with the closest absolute pixel color from the RGB scale for the training data. Here, absolute pixel color means the orthochromatic color, e.g., (255, 0, 0) in RGB for orthochromatic red color. A sample training dataset for the labeling is shown in Table 2. The first four pixel colors in Table 2 are aberrant colors, which the absolute red should replace. The last three pixel colors in Table 2 should be replaced by the absolute blue color. In the model training stage, the following intermediate steps were accomplished: preprocessing the data (handling the pixels), classification of data into more classes, adding new features to the models, analyzing the runtime complexities of the models, performing a methodological comparison of the models. After the accomplishment of all these activities, the test data were used to get an unbiased assessment of the model’s prediction accuracy. Therefore, no shared data existed for the testing set and the training data set. At this stage, the user has to evaluate the model output against the EBSD input image. Evaluation allows the user to validate whether the predefined accuracy was achieved. In case of unsatisfactory results, the prior steps, beginning with data training, need to be revisited so that the root behind causing the model’s under-performance can be identified, and subsequently rectified. The colors in the output image must be predicted accurately. If they are not, it could be due to the following five reasons in generic models: (i) The training dataset did not include the required class. For example, training of the red color might not have been done in the previous training, which appears in the new input image. (ii) There were not sufficient data for a particular class. For example, the total number of training data for the orange color was less than 1000, far less than the 100,000 for blue. This implies that the orange color cannot be identified. It is worth mentioning that a certain color with less data was not a significant problem in our image, since our images resulting from the ML algorithm had good quality, as shown in Figure 5c. (iii) The aberrant colors and the outliers were not fixed in the preprocessing and were used for the training dataset. Such outliers may have negatively influenced the model’s prediction accuracy. (iv) The dataset was not uniform for the different classes (different colors). To ensure there is no bias in the model training, the numbers of training data for all classes should be comparable. There were 60 × 60 pixels for the training of each class (color) in our work. (v) The accuracy of the chosen ML model was low, as not all chosen ML models are suitable for the given task. For an EBSD image, a model with an accuracy larger than 95% would give a satisfactory result. Table 3 lists the accuracies of the applied three models. According to Table 3, the random forest model and the support vector machine model are applicable for the color sortation, and the former have the best quality results. Figure 5c presents the ML predicted images from Figure 5a. After the first application, the model is possibly suitable for direct usage on new untrained data (new image) and reduces the risk of overfitting the data. The previously trained models could at this point be applied to the following images (untrained data), increasing the segmentation’s time efficiency.

#### Ground Truth

Ground truth should be used to calibrate the results from autonomous processes when it is necessary and possible. It also serves to assess the accuracies of different algorithms. It is a difficult task to design a good measurement for the segmentation quality [47]. Still, much work remains for the objective evaluation of segmented outputs [21]. Besides using images with higher accuracy as a baseline for the ground truth [10], Verma et al. presented [48] another approach, which uses mathematical methods to calculate the ground truth. Some authors also took manually segmented tomographic images as the ground truth. Gwet et al. [16] classified the existing performance measurements into two sets, using ground truth and going without. The authors listed several examples/methods to achieve the ground truth in their work. In the current work, it is not necessary to compare the ML result with the ground truth. It is not possible to predict the actual microstructure by any mathematical method in our case. On the one hand, there are no experimental measurements available, which provide an even finer pixel resolution than ≈0.07401 μm per pixel. On the other hand, manual segmentation is too time-consuming for an image with 1080 × 1080 = 1,166,400 pixels to be realized. The widths of grain boundaries, i.e., the slim black contours around grains (e.g., in Figure 5a), are mostly less than ≈0.07401 × 3 = 0.22203 μm. Compared to the mean grain diameter of 4.43 μm in Figure 5a, the inaccuracy is negligible for the ML-identified features.

### 3.3. Postprocessing

**Figure 6:**
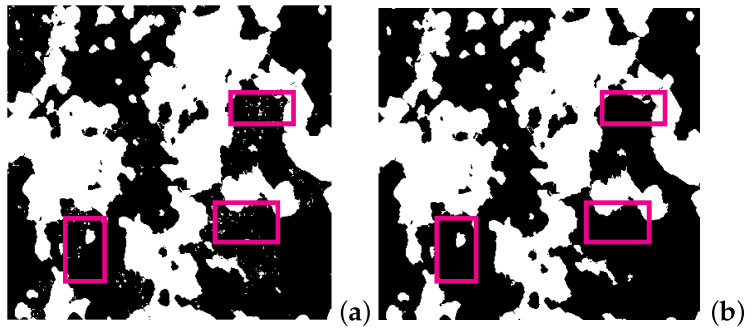
Denoising in postprocessing: (**a**) binary image shown in monotonic color (magenta rectangle in Figure 5d) with disturbances shown in white in black grains and vice versa; (**b**) corresponding image of (**a**) after denoising (magenta rectangle in Figure 5e).

The third step denoised the image resulting from ML. Figure 5c presents the image segmented by ML from the predenoised image Figure 5b. The postprocessing included four substeps. For better visualization of the disturbances and the simplified handling of them, the image was first transformed into an inverse binary image, as shown in Figure 5d. Secondly, white clusters smaller than 25 pixels in diameter (area $\pi \times \frac{25^{2}}{4}\approx 490$) pixels present the Ag grains’ outliers. They are either not part of the grains or interconnected with grain boundaries. These clusters of white pixels (pixel color number 255) were replaced by the surrounding grain pixels (pixel color number 0). Figure 6a, as a zoom-in view of the region enclosed by the rectangle in Figure 5d, depicts the clusters of outlier pixels in the inverse binary image. In the next step, the coordinates of the pixels replaced in the inverse binary image were stored. Figure 6b, as a zoom-in view of the region enclosed by the rectangle in Figure 5e, denotes the denoised image in monotonic color. The three rectangles marked in magenta color in Figure 6a,b denotes the noise’s existence and its disappearance before and after the postprocessing procedure. The corresponding pixel colors in the EBSD image for the final output were replaced by the pixel color of the surrounding grain. Figure 5f illustrates the postprocessed EBSD image compared with the ML-predicted output Figure 5c. On a PC (CPU 3.60 GHz), the whole segmentation time was about 30 min, of which the training time took about two-thirds of the total.

It is worth mentioning that the process in Figure 5 deals with the pixel color. The colors in the final result Figure 5f do not correspond to the color code (Figure 1c) anymore. The aim was to obtain an image presenting local morphologies with much less noise about both Ag grains and SnO${}_{2}$ particles and efficiently prepare the data for meshing. Such pixel colors are irrelevant to the crystallographic orientations. This means EBSD-measured orientations (in digital numbers) are not changed by our image segmentation process. The digital data of measured orientations should be used if an FE simulation with the crystal plasticity requires the input of the initial grain orientation.

### 3.4. Testing and Further Application of Segmentation Algorithms

**Figure 7:**
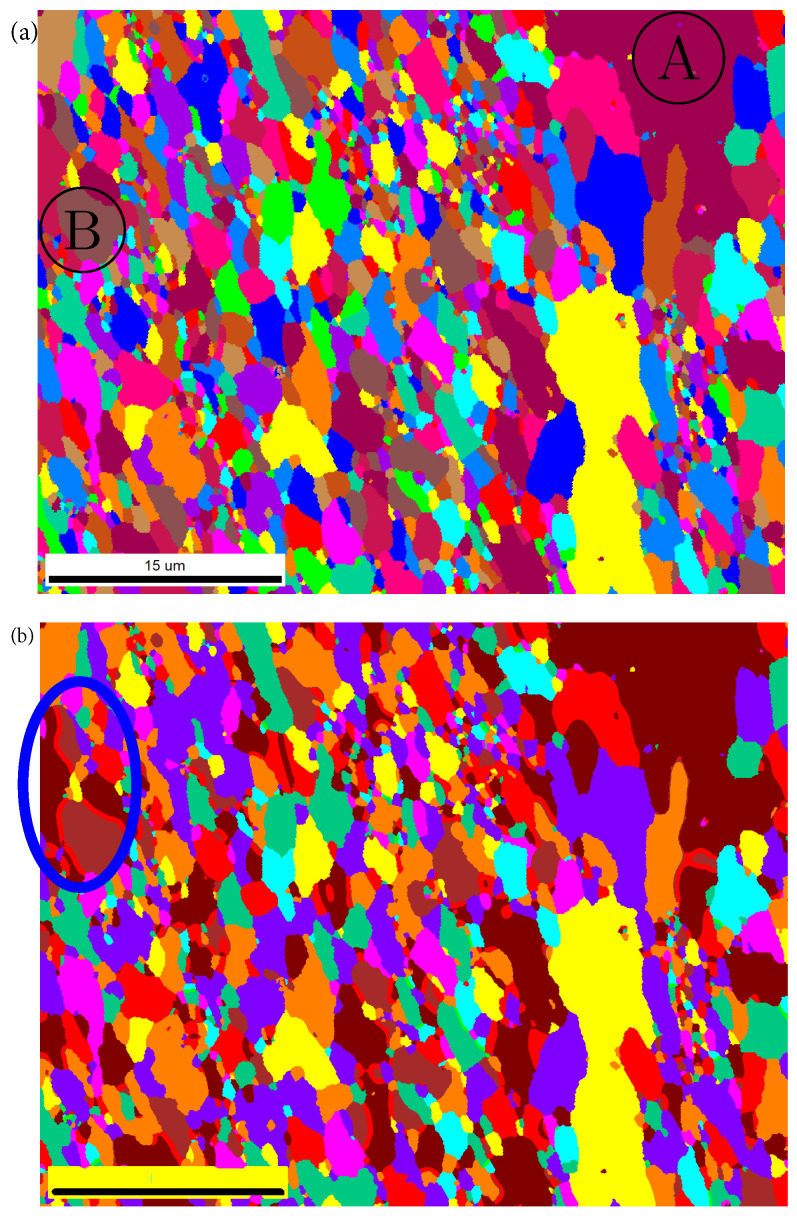
EBSD image from another source for a nanostructured ferritic alloy: (**a**) original EBSD image; (**b**) segmented final result.

EBSD images from another source were segmented to validate our image segmentation method’s general applicability and transferability. For new images, additional colors need to be trained for an increased segmentation quality. Depending on final goals, it is also possible to use the already trained algorithm, i.e., without training it for the new colors. Figure 7a, with a pixel resolution of 1280 × 1000, is an EBSD image for an industrial ferritic alloy, also a kind of ODS steel. This material possesses a mean grain size of about 1 μm. The Cr-oxide and Al-oxide particles have a mean size of about a few to tens of nanometers, and carbides are about about a 30–300 nm. From Figure 7a, Cr-oxide and Al-oxide cannot be identified, since each pixel corresponds to about 37 nm, which is larger than oxides. The carbides might cover 5–8 pixels, making them still too small to be identified. The nano-sized oxide particles with a very high number density show very high thermal stability and can effectively pin the dislocation movement. Such materials often serve in high-temperature environments.

The fine grains and the ultrafine particles lead to clearer grain boundaries than those as shown in Figure 1 (ultrafine particles are invisible in Figure 7a). Still, no noise exists for particles or between boundaries of particles and grains. Such characteristics introduce little noise. In Figure 7a, the brown color is not present; i.e., the algorithm was not yet trained for this color. If the final goal is to mesh the structure, such unknown colors can be assigned to an existing color class (trained color). Figure 7b denotes the segmented result of Figure 7a with the newly trained brown color. Its segmented quality implies that our algorithm is well applicable for such types of materials (EBSD images). As marked in the oval in Figure 7b, some undesired stripes appeared along some grain boundaries. The reason must have been poorly trained color classes. The dark purple marked as Ⓐ in Figure 7a was not trained for and was treated as a kind of brown color, which is different from the brown color marked as Ⓑ. For two neighboring grains with Ⓐ and Ⓑ color, pixel colors on their grain boundaries may be mixed colors from these two colors after training. This pixel color is near to red and categorized as red. In a visualized graph, stripes show up along grain boundaries. The characteristics of the EBSD image as given in Figure 7a are not all the same as those, e.g., in Figure 1 and Figure 2a,b. The differences led to not completely identical considerations for the ML algorithms. Our work emphasized image segmentation for Ag/SnO${}_{2}$ alloys. It means an improvement on the image segmentation for Figure 7a could not be made in this work. In our opinion, it should be easy to remove these stripes along grain boundaries, since only new colors need to be trained.

## 4. Time Efficiency Comparison of Machine Learning and Manual Work

**Figure 8:**
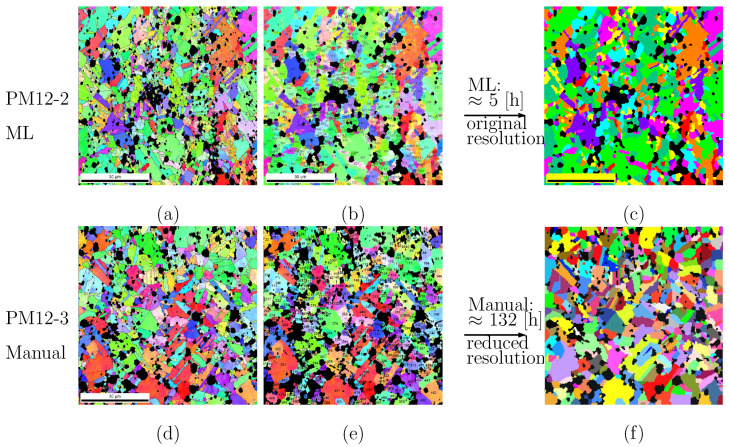
Comparison of image segmenations between autonomous algorithms and manual work for PM12-2 (upper row) and PM12-3 (lower row) Ag/SnO${}_{2}$ composites, respectively: (**a**,**d**) original EBSD images; (**b**,**e**) numbered Ag grains from experiment; (**c**,**f**) segmented images with perfect pixel colors that are ready for meshing.

Figure 8a,d shows the original EBSD images for composites PM12-2 and PM12-3 in the longitudinal direction (Table 1). These present the material’s status after hot extrusion and before tensile loading. The high pixel resolution in Figure 8a,d reveals very tiny grains (about 0.074 μm per pixel), which are far below the average values (Table 1). Such tiny grains are useless for FE simulations. This implies that the original pixel resolution can be coarsened so that less working time is needed for the manual pixel sortation. The ML segmentation of an image with a coarsened pixel resolution (500 × 500 pixels for 80 × 80 $\mu m^{2}$) resulted in output with low quality due to lots of noise and is not shown here. By controlling the element edge length during meshing, the final meshed structures can be comparable for images with identical sizes but different pixel resolutions. Figure 8c (1080 × 1080 pixels) and Figure 8f (300 × 300 pixels) resulted from the autonomous segmentation and the manual work, respectively. They possess the same dimensions but non-identical pixel resolutions. Even for Figure 8f, the element edge length mentioned above was small enough to obtain converged simulation results. This means the pixels’ resolution (Figure 8f) is good enough to achieve meshing convergence. Further refinement of the mesh only caused a negligible difference in the numerically predicted results. The corresponding numbered grains for Figure 8a,d are illustrated in Figure 8b and Figure 8e, respectively. By adjusting the threshold grain size value in the experiment, we controlled the total number of grains in a microstructure cut-out. Figure 8b has 750 Ag grains, whereas Figure 8e has only 513. After recalculating by using the Ag grains’ mean sizes (Table 1), these numbers decreased to 415 and 325 grains, respectively. This implies that a manually segmented image with pixel coarsening can still present a good enough local morphology. Table 4 lists the pixel resolutions in the original images and in the ones during the pixel sortation for the autonomous segmentation and manual work case. It also gives the total number of resulted pixel groups and the time consumed. The name “Resultant groups” in Table 4 indicates that regions with identical pixel colors after ML. Each region covers much more than one grain. E.g., ten resulted (pixel) groups cover a vol% range about [5, 20.5]. The image segmentation done by algorithms took about 0.50 h on a PC (CPU 3.6 GHz). If necessary, the high-performance computation center at the University of Stuttgart can do the same inside a few minutes, depending on the cluster nodes used. After the postprocessing, the resulting image still contained some sparsely distributed noise. An additional 4–5 h of manual work (software GIMP with open access) removed all of them, and the image was then ready for meshing. The commercial meshing software [49] was used, which meshes structures based on pixel colors. It is preferable for a better meshing quality that each Ag grain or particle/cluster contains only one color, the so-called “perfect” color in the current work. Autonomous image segmentation with the application of ML can improve the segmentation quality and the time efficiency by roughly 24 times. For a stack of N EBSD images, there would be an increase in time efficiency of about N × 24 times. Besides EBSD images, the algorithm can segment images from other sources, since the data parsing is pixel-color-based and independent of imaging methods and materials. It is also suitable for various materials, whether with or without pores and voids, and whether liquid or solid. The code deals with three-channel colors (3 × 8${}^{3}$), which means monotonic images should be presented in the same format.

## 5. Applications of Machine Learning Results

In the FE calculation, the particle phase volume/area fraction should have only a slight, and therefore negligible deviation from the actual sample. It means that the particle volume fraction should be checked before meshing. In our work, FE predictions were performed for two composites, PM12-2 and PM12-3 (Figure 8a and Figure 8d, respectively). For the former one, the SnO${}_{2}$ phase attained 16.94 vol.% by the stage “ready for meshing” and 14.73 vol.% for the latter one. For Figure 8f, the particle phase was artificially expanded to 17 vol.%. Figure 8b,e shows the possibility of numbering the Ag grains with a user-defined smallest grain size (from tests). Such figures are useful for FE simulation but not for ML, since the grain boundaries are coarser than those in the original ones (Figure 8a and Figure 8d, respectively). Figure 8c illustrates the segmented images ready for meshing by using an autonomous process plus a small amount of manual work. Figure 8f, also ready for meshing, is the result of purely manual work. FORTRAN code was developed to expand grains based on a grain growth process [50]. In the current work, we only show the meshing process.

**Figure 9:**
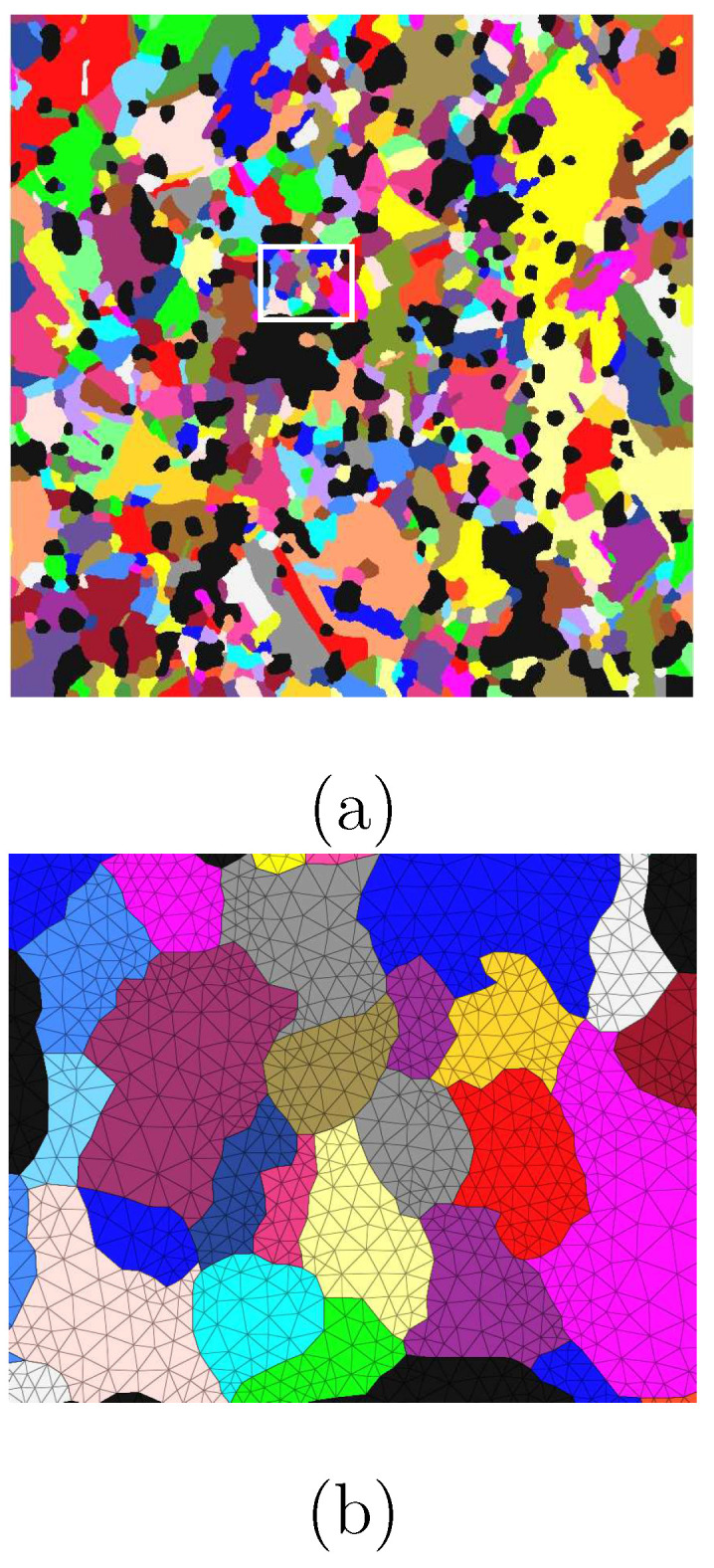
Image (exported from software Hypermesh) with “perfect” pixels from Figure 8b; (**b**) a meshing cut-out corresponding to the white rectangle in (**a**) [49].

Figure 9a illustrates the input image with 17 vol.% SnO${}_{2}$ for meshing. Its original EBSD image is Figure 8b. Figure 9b is the 2D geometrical adaptive meshing for the white rectangle marked in Figure 9a. Since the meshing software SimpleWare ScanIP [49] only meshes 3D structures, a given image should be copied manifold to construct a 3D structure. This software sorts the elements into groups according to pixel colors and delivers geometrical adaptive meshing. After meshing, the information of nodes and elements can be extracted from the surface to obtain a 2D mesh. FORTRAN code did this extraction in our case, and the consumed time was negligible during the preparation for the FE calculation. After this extraction of elements and nodes, the total number of element groups was identical to the pixel color groups. Taking PM12-2 and PM12-3 in Table 4 as examples, there would be 9 and 25 element groups, respectively. Considering the Ag phase, such element groups include element clusters. This implies that not all elements are connected with each other. An element cluster covers at least one Ag grain. If a cluster covers more than one grain, further manual work is necessary to separate the grains by using the numbered grains, as shown in Figure 8b,e. This individual grain identification process is preparation for FE simulations applied with polycrystalline microstructures. Another program was written to automatically identify the individual grains or isolated particles, i.e., neighbors not sharing identical colors. Taking PM12-2 (Figure 8b) as an example, within a few minutes, 638 Ag grains were automatically identified from 9 element groups (excluding the group for SnO${}_{2}$). Some manual work remained in the process of identifying the 750 Ag grains. Depending on the morphology complexity and the working intensity, the manual work took about 1.5–2.0 days to separate the remaining more than 100 Ag grains, as shown in the last column in Table 4. For the simplicity of the initial grain orientation assignment from experiment to FE, the Ag grains’ assigned number should be the same as shown in Figure 8b; i.e., some further manual work was required. E.g., the number of the blue grains in the middle of the lower edge in Figure 8e should be 501. It is pointed out that neighboring pixel groups in the input structure should possess a non-identical color for a 3D geometrical adaptive meshing. Otherwise, the element separation may cause significant inaccuracies of individual grain shapes. For a 2D meshing, separating the pixel groups or element groups should result in a slight and negligible change in accuracy, since the grain morphologies are well recognizable.

**Figure 10:**
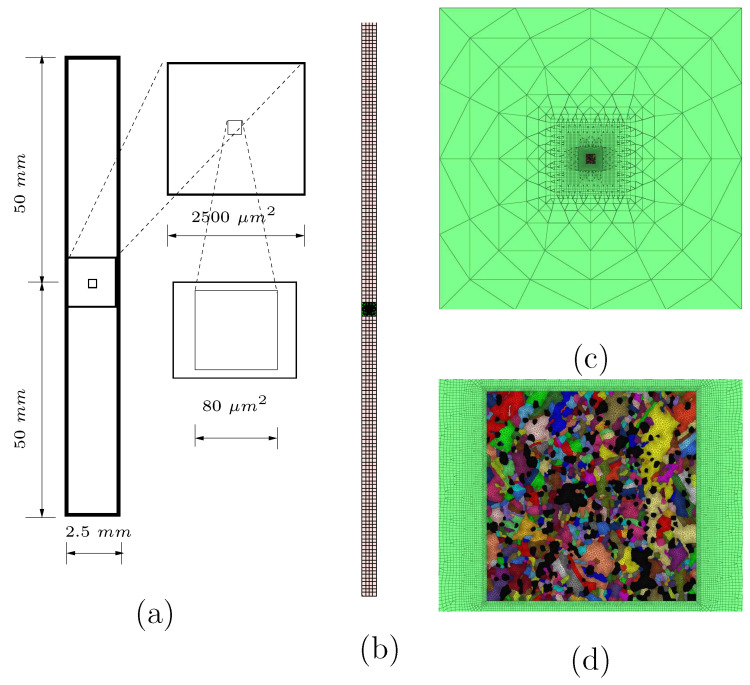
(**a**) Dimensions of micro and macro structures and the transition zone in the two-scale simultaneous FE simulation; (**b**) the meshing of the whole structure in the two-scale simulation; (**c**) meshing in the square-shaped transition zone; (**d**) part of the meshing in the transition zone and the geometrical adaptive meshing of the real microstructure with 750 Ag grains and 222 SnO${}_{2}$ particles (from Figure 8b).

The micro–macro FE simulations were performed simultaneously in our work, i.e., meshing for the macrostructure and the transition zone needed. Hypermesh was used to connect the meshing at the micro–macro-transition level (Figure 10c). Based on our experimental data, Figure 10a illustrates the dimensions for our meshed structures. Regular square-shaped meshing with an edge length of 625 μm was performed on the macro level, as presented in Figure 10b. The meshing in the transition zone should link the meshing on the macro- and micro zones, where triangles are necessary to change the total number of nodes in a fixed dimension. Figure 10c illustrates the meshing of the transition zone; the outer edge of which has the same length as the macro cross-section radius. Figure 10d mainly shows the connection with the micro meshing. There were 61,605 triangle elements in the microstructure, 50,928 of which belonged to the Ag phase. The mean triangle edge length was 0.456 μm for the Ag phase. Six hundred eight square elements were present in the macrostructure, and the transition zone included mixed triangle and rectangle elements. The element types were CGAX3 and CGAX4 (axisymmetry simulaiton in ABAQUS), respectively.

It took about 18–20 working days for manual work to achieve geometrical adaptive meshing for an actual microstructure with 513 grains. Comparatively, it took about 3–4 days with the application of ML for a structure with 750 grains. At the moment, it is not possible to reach a process completely free of manual work for our given task. However, it is well acceptable to finish the task within 3–4 days.

**Figure 11:**
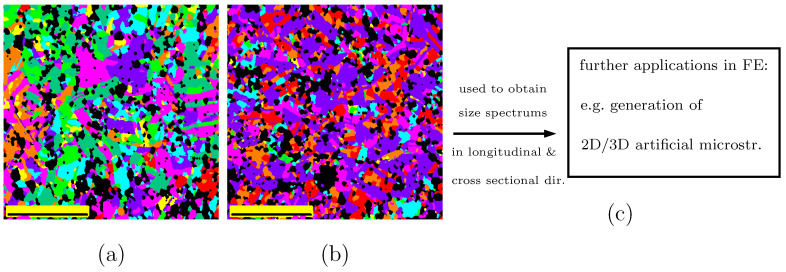
(**a**) Segmented image for Figure 2a; (**b**) segmented image from Figure 2b; (**c**) an example for further applications of ML results in FE simulations. (dir.: direction, microstr.: microstructure).

A further application based on our ML results could be the provision of experimental data to generate 2D/3D artificial microstructures. Schneider et al. [51] presented a numerical method to generate hierarchical 2D/3D Poisson–Voronoi structures. The generation of artificial microstructures will be reported in our future work to compare the boundary conditions’ influences on the deformation behavior. Figure 11a presents the segmented results of the original images shown in Figure 2a for the longitudinal direction and Figure 11b for the cross-sectional direction. After using an available program to separate neighboring grains not sharing the same color in Figure 11a, the remaining individual grains would be identified with a slight amount of additional manual work. It would then be easy to calculate the grain/particle areas and plot their size distributions. Furat et al. [31] showed the grain boundary identification from monotonic tomographic images by using ML. Nevertheless, their method cannot be directly used in our colored EBSD images. If an algorithm can identify the grain boundaries in the EBSD images, the above-mentioned manual work to identify neighboring grains sharing identical colors would not be necessary. However, this was not our primary goal and has not been implemented in our work yet.

For multiscale investigation of material behaviors, ML results can also contribute to the bridging/linking of physical or mechanical variables on different dimensions. Based on autonomous segmented results, e.g., Figure 5f, Figure 8c and Figure 11a,b, our subsequent work would statistically calculate the mean size of selected grains. Such grains possess approximately the same orientation and would have identical color after the digital segmentation. They together form a group called the “orientation group”. E.g., Figure 5f possesses eight orientation groups, i.e., eight different colors for the Ag phase. All the grain orientations in each orientation group would have a resulting orientation (through tensor addition with grain area/volume weighting factor). This means the microstructure cut-out (Figure 5f) possesses eight above-mentioned “resulted orientations”, which can be used in discrete dislocation dynamic (DDD) simulations. In the DDD simulations, the initial randomly distributed dislocations are assumed as several finite-length segments. Following the interaction rules (e.g., Peach–Koehler forces, mobility laws) and considering mechanisms such as the cross-slip and the junction formation, a 3D simulation of the dislocation motion can be achieved. Such numerical calculations can establish the relation between local Young’s moduli (local yield stresses) and the critical resolved shear stress (CRSS) on the submicron level. The evolution of resolved shear stress can also be predicted. Nanoindentation tests can deliver curves of the local Young’s moduli and drilling depths, i.e., local elasticities on different dimensions. Measured CRSSs from micropillar tests can calibrate the numerical one. For in-depth information on DDD and its usage in simulations, on can refer to, e.g., [52,53]. Po et al. [54] reported a review of the DDD method for numerical investigations of plasticity in crystals. Molnar et al. [55] published some previous work on DDD simulations. The submicron dimension covered by DDD is the upper limit for molecular dynamics simulations [53] on the nanoscale. At the micro-level, the crystal plasticity FE simulation can predict the evolution of the resolved shear stress; e.g., see our previous work [56,57]. To link the nanoscale and microscale, the predicted CRSS from DDD can be used as the initial value in the crystal plasticity FE simulations. The numerically calculated evolution of the resolved shear stress can be compared between DDD and FE simulations.

## 6. Conclusions

In order to improve the time efficiency of the data preparation for multiscale FE simulations, this work adopted ML for a digital image segmentation process to extract morphological features of real polycrystalline microstructures. We developed our own Python algorithms with the usage of inherent libraries/packages. Our autonomous process dealing with EBSD images includes three steps, namely, preprocessing, ML, and postprocessing. Three supervised ML methods—decision tree, random forest, and support vector machine—are applied, and their accuracies were compared. Each of our examples contains no less than a few hundred grains and more than 1 million data samples (pixels). Image segmentation results gained using manual segmentation are also presented to quantify the improved efficiency of the new wrokflow. A coarsened pixel resolution, 1080 × 1080 pixels, scaled down to 300 × 300 in our case, enables the realization of the manual image segmentation. It is worth mentioning that no logical difference exists between the segmentation of 2D and tomography-based 3D microstructures. As a further step in preparation for FE simulation and as an ML application, the geometrically adaptive meshing was also presented. ML results can also contribute to the scale bridging for physical/mechanical properties, such as CRSS on the nanoscale and microscale. From the achieved results, the following conclusions can be drawn:

- It is essential to execute the denoising before and after ML to achieve a good segmentation quality, i.e., preprocessing and postprocessing.
- The digital image segmentation process with ML possesses a high quality for the detailed feature extraction. Such a high quality is hardly reachable using manual segmentation, since the handling of millions of data samples is too time-consuming to be realized.
- Two supervised ML methods, random forest and support vector machine, showed accuracies higher than 99.8%.
- The autonomous ML process improves the segmentation’s time efficiency for polycrystalline EBSD images by a factor of no less than 24 times for a single image. For serial works, the time efficiency would be improved multiplicatively.
- From an original polycrystalline microstructure to the final meshed one ready for FE calculation, this efficiency is improved approximately six times, since some manual work is inevitable.

## Figures and Tables

**Table 1:** A list of essential properties of the two MMCs used in the current work [27].

Material	Oxide wt.%	Oxide Vol.%	Grain Mean Size D${}_{50}$ [μm]	Oxide Mean Size d${}_{50}$ [μm]	$R_{p0.2}$ [MPa]
PM12-2	12	17	4.43 ± 0.5	0.56 ± 0.16	118
PM12-3	12	17	5.00 ± 0.5	0.93 ± 0.20	106
Ag 99.97	-	-	-	-	43

**Table 2:** A sample of the labelling for the input EBSD image data, which was used as the training dataset.

R	G	B	Label
245	5	10	1
240	30	10	1
235	0	35	1
250	5	5	1
25	5	254	2
2	3	244	2
10	8	252	2

**Table 3:** An example to show the prediction accuracies of the three ML models.

Model	Decision Tree		Support Vector Machine	Random Forest
	Gini	Entropy		
Accuracy	85.81%	93.30%	99.84%	99.86%

**Table 4:** Time consuming comparison of image segmentations executed by ML and manual work.

Type	Material/ Ag Grain Nr.	Pixel Resolution		Resulted Groups	Pixel Segm. [h]	Perfect Pixels [h]	Meshing Until Ready for FE Cal. [h]
		Original	Working on				
ML	PM-12-2/750	1080 × 1080	1080 × 1080	9	≈0.5	≈4.0–5.0	≈15.0
Manual	PM-12-3/513	1080 × 1080	300 × 300	25	≈120.0	-	≈12.0

## Data Availability

Data are contained within the manuscript.

## References

[1] Agrawal A., Choudhary A. (2016). Perspective: Materials informatics and big data: Realization of the “fourth paradigm” of science in materials science. APL Mater..

[2] Samuel A.L. (1959). Some Studies in Machine Learning Using the Game of Checkers. Comput. Sci. (IBM J. Res. Dev.).

[3] Wang H., Ji Y., Li Y. (2020). Simulation and design of energy materials accelerated by machine learning. WIREs Comput. Mol. Sci..

[4] Kannan S., Nalini G., Gurusamy V. (2014). Review on Image Segmentation Techniques. https://www.researchgate.net/publication/273127438.

[5] Jing X., Yan Z., Witold P. (2018). Security data collection and data analytics in the internet: A survey. IEEE Commun. Surv. Tutor..

[6] Faggella D. What Is Machine Learning. https://emerj.com/ai-glossary-terms/what-is-machine-learning/.

[7] Butler K.T., Davies D.W., Cartwright H., Isayev O., Walsh A. (2018). Machine learning for molecular and materials science. Nature.

[8] Wu W., Su Q. (2018). Applying machine learning to accelerate new materials development. Sci. Sin. Phys. Mech. Astron..

[9] Wei J., Chu X., Sun X., Xu K., Deng H., Chen J., Wei Z., Lei M. (2019). Machine learning in materials science. InfoMat.

[10] Ronneberger O., Fischer P., Brox T. (2015). U-Net: Convolutional Networks for Biomedical Image Segmentation. Medical Image Computing and Computer-Assisted Intervention.

[11] Abiodun O.I., Jantan A., Omolara A.E., Dada K.V., Umar A.M., Linus O.U., Arshad H., Kazaure A.A., Gana U., Kiru M.U. (2019). Comprehensive Review of Artificial Neural Network Applications to Pattern Recognition. IEEE Access.

[12] Ramprasad R., Batra R., Pilania G., Mannodi-Kanakkithodi A., Kim C. (2017). Machine learning in materials informatics: Recent applications and prospects. npj Comput. Mater..

[13] Bock F.E., Aydin R.C., Cyron C.J., Huber N., Kalidindi S.R., Klusemann B. (2019). A Review of the Application of Machine Learning and Data Mining Approaches in Continuum Materials Mechanics. Front. Mater..

[14] Meng T., Jing X., Yan Z., Pedrycz W. (2020). A survey on machine learning for data fusion. Inf. Fusion.

[15] Jaiswal S., Pandey M.K., Rathore V., Dey N., Piuri V., Babo R., Polkowski Z., Tavares J. (2021). A Review on Image Segmentation. Rising Threats in Expert Applications and Solutions, Advances in Intelligent Systems and Computing 1187.

[16] Gwet D.L.L., Otesteanu M., Libouga I.O., Bitjoka L., Popa G.D. (2018). A Review on Image Segmentation Techniques and Performance Measures. Int. J. Comput. Inf. Eng..

[17] Kuruvilla J., Sukumaran D., Sankar A., Joy S.P. A review on image processing and image segmentation. Proceedings of the 2016 International Conference on Data Mining and Advanced Computing (SAPIENCE).

[18] Jain R., Sharma R.S., Yadav N. (2019). Image Segmentation Through Fuzzy Clustering: A Survey. Harmony Search and Nature Inspired Optimization Algorithms, Advances in Intelligent Systems and Computing 741.

[19] Anjna E., Rajandeep Kaur E. (2017). Review of Image Segmentation Technique. Int. J. Adv. Res. Comput. Sci..

[20] De S., Bhattacharyya S., Chakraborty S., Dutta P. (2016). Hybrid Soft Computing for Multilevel Image and Data Segmentation.

[21] Pal N.R., Pal S.K. (1993). A review on image segmentation techniques. Pattern Recognit..

[22] Lee L.K., Liew S.C., Thong W.J., Sulaiman H.A. (2015). A Review of Image. Advanced Computer and Communication Engineering Technology.

[23] Chowdhary C.L., Acharjya D.P. (2020). Segmentation and Feature Extraction in Medical Imaging: A Systematic Review. Procedia Comput. Sci..

[24] Kollem S., Reddy K.R.L., Rao D.S. (2019). A Review of Image Denoising and Segmentation Methods Based on Medical Images. Int. J. Mach. Learn. Comput..

[25] Zhou T., Ruan S., Canu S. (2019). A review: Deep learning for medical image segmentation using multi-modality fusion. Array.

[26] Wasserbäch W., Skrotzki W. (2019). Microstructure and texture development in oxide-dispersion strengthened silver rods processed by hot-extrusion. Materialia.

[27] Wasserbäch W., Skrotzki W., Chekhonin P. (2020). Strengthening of ODS silver wires. Materialia.

[28] Lutz O., Behrens V., Wasserbäch W., Franz S., Honig T., Späth D., Heinrich J. Improved Silver/Tin Oxide Contact Materials for Automotive Applications. Proceedings of the 24th International Conference on Electrical Contacts (ICEC).

[29] (2002). Powder Metal Technologies and Applications, Powder Metallurgy Electrical Contact Materials. ASM Handb..

[30] Huang J., Strunk H.P., Wasserbäch W., Franz S. (2009). Internally oxidized silver contact materials-a case for the elastoplasticity of an inhomogeneous body. Cryst. Res. Technol..

[31] Furat O., Wang M., Neumann M., Petrich L., Weber M., Krill C.E., Schmidt V. (2019). Machine Learning Techniques for the Segmentation of Tomographic Image Data of Functional Materials. Front. Neurosci..

[32] Pütz F., Henrich M., Roth A., Könemann M., Münstermann S. (2020). Reconstruction of Microstructural and Morphological Parameters for RVE Simulations with Machine Learning. Procedia Manuf..

[33] Sharma R., Chelladurai I., Orme A.D., Miles M.P., Giraud-Carrier C., Fullwood D.T. (2018). A step towards intelligent EBSD microscopy: Machine-learning prediction of twin activity in MgAZ31. J. Microsc..

[34] Bostanabad R., Bui A.T., Xie W., Apley D.W., Chen W. (2016). Stochastic microstructure characterization and reconstruction via supervised learning. Acta Mater..

[35] Li Y., Holmedal B., Liu B., Li H., Zhuang L., Zhang J., Du Q., Xie J. (2021). Towards high-throughput microstructure simulation in compositionally complex alloys via machine learning. Calphad.

[36] Tercan H., Guajardo A., Heinisch J., Thiele T., Hopmann C., Meisen T. (2018). Transfer-Learning: Bridging the Gap between Real and Simulation Data for Machine Learning in Injection Molding. Procedia CIRP.

[37] Tawfik S.A., Isayev O., Spencer M.J.S., Winkler D.A. (2020). Predicting Thermal Properties of Crystals Using Machine Learning. Adv. Theory Simul..

[38] Baturynska I., Semeniuta O., Martinsen K. (2018). Optimization of Process Parameters for Powder Bed Fusion Additive Manufacturing by Combination of Machine Learning and Finite Element Method: A Conceptual Framework. Procedia CIRP.

[39] Johnson D. (2020). Supervised vs. Unsupervised Learning: Key Differences. https://www.guru99.com/supervised-vs-unsupervised-learning.html.

[40] Jaiswal S. (2018). Regression vs. Classification in Machine Learning. https://www.javatpoint.com/regression-vs-classification-in-machine-learning.

[41] Jaiswal S. (2018). Difference between Supervised and Unsupervised Learning. https://www.javatpoint.com/difference-between-supervised-and-unsupervised-learning.

[42] Shalev-Shwartz S., Ben-David S. (2014). Understanding Machine Learning: From Theory to Algorithms.

[43] Johnson W. (2016). Decision Tree Flavors: Gini Index and Information Gain. http://www.learnbymarketing.com/481/decision-tree-flavors-gini-info-gain/.

[44] Breiman L. (2001). Random Forests. Mach. Learn..

[45] Gandhi R. (2018). Support Vector Machine—Introduction to Machine Learning Algorithms. https://towardsdatascience.com/support-vector-machine-introduction-to-machine-learning-algorithms-934a444fca47.

[46] Abilash R. (2018). Applying Random Forest (Classification)—Machine Learning Algorithm from Scratch with Real Datasets. https://medium.com/@ar.ingenious/applying-random-forest-classification-machine-learning-algorithm-from-scratch-with-real-24ff198a1c57.

[47] Celebi M.E., Lecca M., Smolka B. (2015). Color Image and Video Enhancement.

[48] Verma D., Agarwal H., Aggarwal A.K., Yadav N., Yadav A., Bansal J.C., Deep K., Kim J.H. (2018). Palmprint Matching based on Normalized Correlation Coefficient and Mean Structural Similarity Index Measure. Advances in Intelligent Systems and Computing, 741, Harmony Search and Nature Inspired Optimization Algorithms.

[49] (2017). Simpleware ScanIP. https://www.synopsys.com/simpleware/software/scanip.html.

[50] Schneider Y., Wasserbäch W., Schmauder S., Zhou Z., Zielke R., Tillmann W. (2021). A Numerical Method to Improve the Representativeness of Real Microstructure Cut-Outs Applied in Finite Element Simulations. Crystals.

[51] Schneider Y., Weber U., Wasserbäch W., Zielke R., Schmauder S., Tillmann W. (2020). A numerical method for the generation of hierarchical Poisson Voronoi microstructures applied in micromechanical finite element simulations part I: Method. Comput. Mech..

[52] Lepinoux J., Kubin L.P. (1987). The dynamic organization of dislocation structures: A Simulation. Scr. Metall..

[53] Kubin L.P., Canova G., Condat M., Devincre B., Pontikis V., Bréchet Y. (1992). Dislocation Microstructures and Plastic Flow: A 3D Simulation. Solid State Phenom..

[54] Po G., Mohamed M.S., Crosby T., Erel C., El-Azab A., Ghoniem N. (2014). Recent Progress in Discrete Dislocation Dynamics and Its Applications to Micro Plasticity. Met. Mater. Soc..

[55] Molnar D., Weber U., Binkele P., Rapp D., Schmauder S. (2015). Prediction of macroscopic damage behaviour of precipitation strengthened steels via multiscale simulations. GAMM-Mitt.

[56] Schneider Y., Bertram A., Böhlke T., Hartig C. (2010). Plastic deformation behaviour of Fe-Cu composites predicted by 3D finite element simulation. Comput. Mat. Sci..

[57] Schneider Y., Bertram A., Böhlke T. (2013). Three-dimensional Simulation of Local and Global Behaviour of *α*Fe-Cu Composites under Large Plastic Deformation. Tech. Mech..

